# Do Helpful Mothers Help? Effects of Maternal Scaffolding and Infant Engagement on Cognitive Performance

**DOI:** 10.3389/fpsyg.2019.02661

**Published:** 2019-11-28

**Authors:** Kaili Clackson, Sam Wass, Stanimira Georgieva, Laura Brightman, Rebecca Nutbrown, Harriet Almond, Julia Bieluczyk, Giulia Carro, Brier Rigby Dames, Victoria Leong

**Affiliations:** ^1^Department of Psychology, University of Cambridge, Cambridge, United Kingdom; ^2^Department of Psychology, University of East London, London, United Kingdom; ^3^Division of Psychology, School of Social Sciences, Nanyang Technological University, Singapore, Singapore

**Keywords:** social interaction, maternal responsiveness, engagement, object search, scaffolding (teaching technique)

## Abstract

Infants are highly social and much early learning takes place in a social context during interactions with caregivers. Previous research shows that social scaffolding – responsive parenting and joint attention – can confer benefits for infants’ long-term development and learning. However, little previous research has examined whether dynamic (moment-to-moment) adaptations in adults’ social scaffolding are able to produce immediate effects on infants’ performance. Here we ask whether infants’ success on an object search task is more strongly influenced by maternal behavior, including dynamic changes in response behavior, or by fluctuations in infants’ own engagement levels. Thirty-five mother-infant dyads (infants aged 10.8 months, on average) participated in an object search task that was delivered in a naturalistic manner by the child’s mother. Measures of maternal responsiveness (teaching duration; sensitivity) and infant engagement (engagement score; visual attention) were assessed. Mothers varied their task delivery trial by trial, but neither measure of maternal responsiveness significantly predicted infants’ success in performing the search task. Rather, infants’ own level of engagement was the sole significant predictor of accuracy. These results indicate that while parental scaffolding is offered spontaneously (and is undoubtedly crucial for development), in this context children’s endogenous engagement proved to be a more powerful determinant of task success. Future work should explore this interplay between parental and child-internal factors in other learning and social contexts.

## Introduction

Finding a hidden object involves a number of cognitive processes which develop significantly during the first year of life – including the ability to pay attention to the object as it is hidden, to remember where it is stored while it cannot be seen, and to inhibit the urge to perseverate in reaching to a previously stored location (Munakata, 1998). For this reason, hiding and finding games such as the A-not-B task and variants thereof have been extensively used by developmental psychologists to measure the development of executive functions (e.g., Diamond et al., 1994; Bell and Adams, 1999; Thelen et al., 2001; Marcovitch et al., 2016). The A-not-B task involves the researcher hiding an object in one of two containers in view of the infant (location A), and the infant is then monitored to see if s/he searches for the toy in the correct location. When the infant has successfully located the toy at location A, in subsequent trials the object is hidden in the other location (B), again in sight of the infant, and the infant is then allowed to search for the toy again. The task is usually delivered by a researcher in a standardized way so as to minimize any influence from variations in the performance of the demonstrator delivering the task. However, much of infants’ natural learning occurs in social contexts, often involving a delicate “dance” between infant and caregiver where each evaluates the actions and motivations of the other, and moderates their behavior from moment to moment accordingly. Here, we investigate how infants perform on an object search task when it is embedded in a naturalistic social context, such as a game between mother and infant. Specifically, we asked whether the infant’s performance is primarily moderated by social factors relating to the mother’s delivery of the task, or factors internal to the infant such as attention and engagement. The remainder of the introduction examines how research in different areas of infant development predicts differing answers to this question.

### Maternal Scaffolding May Improve Infant Performance

Research suggests that in naturalistic contexts, where information is being passed from a mother to her child, the behavior of the mother will affect how successfully the infant receives the information transmitted. From around a year, visual attention (crucial for object search tasks) is moderated by social context, with infants looking longer toward a toy during free play if a parent is also attending to the toy (Yu and Smith, 2016). Research measuring neural activity (e.g., electroencephalography, EEG) during social scenarios has shown that when 9-month-old babies engage in joint attention with an adult who directs their attention to an object, the mid-latency negative component (Nc) of the infant event-related potential (ERP), an index of attentional processes, is enhanced during the processing of the object (Striano et al., 2006). Infants’ responses to adults’ visuospatial attentional cueing can also be improved with attention training, showing that the development of visual attention (in a joint attention context) is mediated by an interaction between internal cognitive abilities and external factors (Forssman and Wass, 2017). The ability to engage in joint attention is closely related to an infant’s social and intellectual development. For example, engagement in joint attention at 12 months has been linked to improved language outcomes at 24 months (Morales et al., 2000; Mundy et al., 2007) and infants who engage in more mutual gaze at 5 months show superior visual attention control at 11 months (Niedźwiecka et al., 2017). Joint attention behavior at 12 months also predicts fewer parental reports of negative behavior in areas such as aggression, defiance and impulsivity at 30 months (Vaughan Van Hecke et al., 2007). Since visual attention to the correct object at the correct time (i.e., as the toy is hidden in a particular location) is crucial for success in object search tasks, it is reasonable to expect that parental scaffolding of infants’ attention would be beneficial.

Similarly, studies in responsive parenting have shown that when responses to an infant’s bids for attention are prompt, appropriate, and tailored both to the specific situation and to the child’s developmental level, infants show more positive developmental outcomes, particularly in the area of language (Landry et al., 1997; Tamis-LeMonda et al., 2001; Paavola et al., 2005; Bornstein et al., 2008; Vally et al., 2015). While responsive parenting and the infant’s own willingness to initiate interactions have been shown to contribute separately to the development of early communication skills (Paavola et al., 2005), the two are often closely linked. Parents’ responsive behavior occurs, by definition, in response to some act on the part of the child, with more communicative infants providing parents with more opportunities to respond. Infants and caregivers directly influence each other in this area, as direct eye contact from adults elicits more infant vocalizations (Leong et al., 2017), and the way that an infant responds to caregivers directly affects the quality of care the infant receives (Vallotton, 2009). Accordingly, it is clear that in any interactive situation between mother and child, the behavior of each does not occur in isolation, but is heavily contingent upon the behavior of the other. The infant enjoys more positive outcomes when the mother engages the infant in joint attention and adapts her behavior in response to her infants’ by changing her style and pace of interaction. Therefore, if the social aspects of a task are enhanced (i.e., the task is delivered naturalistically by the child’s mother, rather than in a standardized fashion by an experimenter), then infants should perform better on trials where the mother shows higher levels of responsive behavior to her infant. For simplicity, this will be referred to as the *maternal scaffolding hypothesis.*

### Infant Performance May Rely on Factors Internal to the Infant

Evidence from previous studies suggests that, for object search tasks in particular, social information may in fact lead to *higher* error rates. Topál et al. (2008) looked at 10 month-old’s perseverative search errors in an A-not-B object search task and showed that error rates were substantially reduced when communicative or social aspects of the task were removed. When the experimenter faced away from the infant, making no eye contact and not communicating with the infant in any way while hiding the toy, the proportion of infants showing perseverative errors was significantly reduced from 86% to 43%, and when the experimenter was hidden behind a curtain so that only the movements of the objects could be seen, the proportion of errors fell even lower (36%). Topál et al. (2008) conclude that the use of ostensive-referential signals (eye contact, calling infant’s name, pointing, etc.) can trigger an assumption in the infant that the information he is being presented with is generalizable, rather than episodic, so that the infant interprets the hiding at A as showing a generalizable property of the object such that “toys such as this are found in location A.” As each toy was hidden four times in location A prior to being hidden in location B, the experimental design particularly strengthened the interpretation that this toy is *always* found at location A. Nine month old infants have also been shown to retain qualitatively different information about novel objects in differing social contexts, focusing attentional resources on an object’s identity (at the expense of location) in a communicative context, and on an object’s location (at the expense of identity) in a non-communicative context (Yoon et al., 2008).

Such findings demonstrate that, for object search tasks, infant performance can in fact be hindered by the availability of social cues in cases where the social cues can be misleading. In cases where the infant chooses the correct location despite these social cues, their success in finding a hidden object must, therefore, rely on factors internal to the infant. Considerable research has sought to identify what such infant internal factors might be. Short-term memory has been shown to play a role, as infants’ performance is affected by the duration of the delay between hiding and being allowed to reach for the hidden object. When there is no delay, perseverative errors are rare, however, performance deteriorates when the delay is increased (Diamond, 1985; Clearfield et al., 2009). Inhibitory control is also necessary for success on the task, as infants need to inhibit the repetition of an action that was successful in the past (seeking the toy at location A). Berger (2004) used a parent-seeking task similar to the A-not-B task to show that even 13 month old children had difficulty in inhibiting a previously successful response when task demands were increased. When children walked toward parents on flat ground they did not perseverate on B trials. However, when they were placed on a platform so that they had to descend a staircase to reach the parent, they had more difficulty in inhibiting repeated responses that were no longer appropriate and showed locomotor perseveration on 25% of trials. While both memory and inhibitory control are expected to improve over development with maturation of the prefrontal cortex, here we choose to focus on another infant internal factor that can affect performance on shorter, moment-to-moment timescales: infants’ engagement in the task. We defined engagement operationally as the degree to which infants displayed positive affect and body language, interest, attention and goal-directed behavior whilst performing the task. We reasoned that while executive functions such as memory and inhibition would remain stable over the course of the testing session, infants’ engagement levels were likely to fluctuate, allowing us to measure the effect of this infant internal factor on search performance. If it is the case that performance on the task is driven by infant internal factors, then irrespective of the effort that mothers put into social interaction, infants’ performance will be determined primarily by their own level of engagement. This will be referred to as the *infant internal hypothesis.*

### Predictions

The two fields of research discussed above make differing predictions as to how an infant might perform on a naturalistic object search task.

#### Maternal Scaffolding Hypothesis

If the social interaction between a mother and her child improves her child’s chance of correctly locating the toy, this would be shown by a significant and positive relationship between the mother’s adaptive delivery of the task and the child’s accuracy on the task.

#### Infant Internal Hypothesis

If the child’s performance on the task is due to infant internal factors, there may be no relationship between the mother’s adaptive delivery of the task and the infant’s success, with performance being predicted solely by engagement factors internal to the infant. Furthermore, if the social interaction between a mother and her child actually hinders accurate localization of the toy, this may be shown by a negative relationship between the mother’s adaptive delivery of the task and the child’s accuracy.

## Materials and Methods

### Participants

Thirty-five^1^ mother-infant pairs participated in the study. The infants showed a 19M/16F gender split. Infants were aged between 274–390 days (9.0–12.8 months) with a mean age of 327 days (10.8 months) (SD: 35 days) and all received at least 50% of their language input in English (and had done so for at least 3 months prior to taking part in the study). All infants were developing normally with no neurological problems or diagnoses of developmental difficulty or delay. Participants were recruited through flyers at local baby groups and nurseries, and via an advert in the local National Childbirth Trust magazine. The study received ethical approval from the [blinded] Psychology Research Ethics Committee (PRE.2016.029, project name [blinded]), and all methods were carried out in accordance with the relevant guidelines and regulations. Parents provided written informed consent on behalf of their infants.

### Materials

**FIGURE 1:**
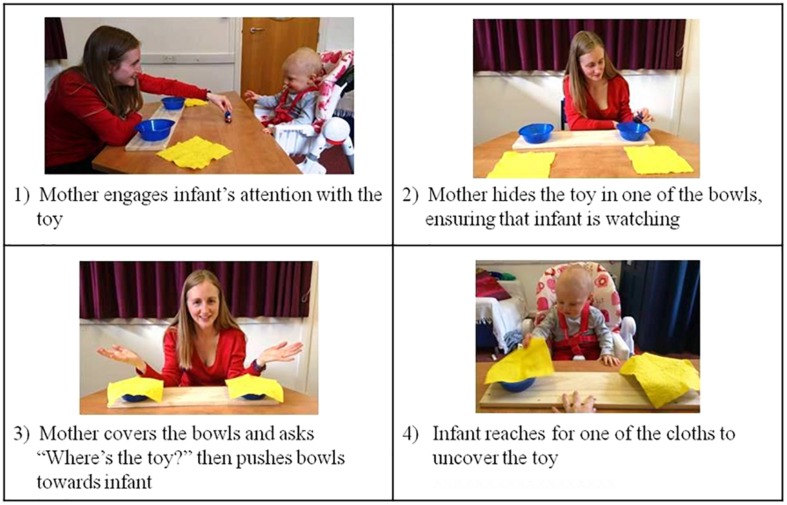
Illustration of the object search task. Written consent was provided for the use of these images.

The object search task involved two plastic bowls, two covering cloths and a set of small toys. The bowls were attached to a base so as to keep them at a constant equal distance of 32 cm from each other, and to avoid the infant knocking them over or off the table. The demo toy (used to show the infant how the “game” works) was a train carriage with moving parts, and experimental toys were a plastic dinosaur, a toy steam train, a circular rattle and a rubber finger puppet with dangly eyeballs. To keep the infant’s interest and make each trial visually different, two different sets of cloths (one set yellow and one set striped red, white and blue) and two sets of bowls (one set pink and one set blue) were used. Cloths were swapped every trial, and bowls every two trials in a counterbalanced order across participants. Figure 1 illustrates the experimental set up.

### Object Search Task

In this task a toy was hidden by the mother in one of two locations in sight of the infant, and the child was then given the opportunity to look for and find the toy. During the task, the infant was seated in a highchair across a table from the mother, who was at arm’s reach distance. As shown in Figure 1, for each experimental trial, the mother placed two bowls on the table in front of her (out of her infant’s reach), then attracted her infant’s attention to a small toy, and placed it in one of the bowls, ensuring that the infant’s gaze followed the toy into the bowl. The mother then simultaneously covered both bowls with cloths and held her hands spread out and palms up while asking “Where’s the toy?” The mother then moved the two covered bowls across the table toward the infant, ensuring that each bowl was equidistant from the child. The infant’s reaching actions were recorded live by the experimenter and verified by video coding afterward.

Prior to commencing the experimental trials, the mother demonstrated four trials to the infant. During these demo trials, the hiding procedure was the same as for experimental trials, but after asking “Where’s the toy?” the mother would proceed to lift the cloths in turn and “find” the toy herself. The location of the toy alternated for each demo trial. On finding the toy she would exclaim excitedly and show her infant the toy. After the demonstration trials, the toy was changed and the infant completed up to 16 experimental trials, with the toy being changed every four trials to maintain the infant’s interest (i.e., four trials per toy). The order of the four experimental toys, and which bowl the toy was first hidden in was counterbalanced across infants following a Latin square design. An example trial order is shown in Table 1. Across the four toys, two toys had a starting hiding position on the right and the other two toys had a starting hiding position on the left to avoid a side bias caused by each toy being first hidden on the same side. Subsequent trials alternated the hiding location of the same toy from left to right. This meant that of the 15 trials following the first trial, 12 involved the toy being hidden on the opposite side to the previous trial, and three involved the toy being hidden on the same side as the previous trial.

**TABLE 1:** An example trial order.

**Trial No.**	**Toy**	**Bowl toy is hidden in**
Demo 1	Demo toy	Left
Demo 2		Right
Demo 3		Left
Demo 4		Right
1	Toy 1	Right
2		Left
3		Right
4		Left
5	Toy 2	Left
6		Right
7		Left
8		Right
9	Toy 3	Right
10		Left
11		Right
12		Left
13	Toy 4	Left
14		Right
15		Left
16		Right

This procedure differed from the standard A-not-B task as we were specifically interested in the role of social factors on the infant’s accuracy, and this procedure allowed us to collect data from every trial (rather than only focusing on “B” trials), meaning that a larger number of data points could be collected from each infant.

For each trial, the experimenter recorded the infant’s valid response in two categories: (1) the infant looked for the toy in the correct bowl, or (2) the incorrect empty bowl. The infants’ response was determined by the first cloth/bowl that was touched following an arm reach. If the infant touched both cloths at the same time, failed to give a response (didn’t touch either cloth), or was guided by the mother (such as mother pointing to the correct bowl), this was recorded as an invalid response. Invalid responses were excluded from the analysis and accounted for 3.9% of the full dataset. The infant’s responses were recorded live during the task by the experimenter, and later validated by a separate coder when reviewing and coding the video of the session (section “Data Processing and Analysis”).

To ensure that all mothers followed the same procedure in delivering the task, mothers were sent detailed instructions explaining the stages shown in Figure 1 before attending their lab visit. On arrival at the lab, the experimenter again explained and demonstrated the stages of the game. The mother was reminded that she should ensure that the infant saw the toy in the bowl before it was covered. To ensure that the data would contain sufficient variations in maternal delivery style, mothers were encouraged to make the game enjoyable for her child and to present the task in a natural, engaging manner. During the task the experimenter was seated on a low stool at the side of the table so as to prompt the mother regarding which side the toy should be hidden on, and to ensure that the correct protocol was followed. Each session was recorded by three video cameras. One camera recorded a view of the mother, and the area of the table in front of her. A second camera recorded the infant and the area of the table in front, with some overlap so that the center area of the table was visible to both cameras. These two main cameras were used for video coding of events. A third camera was placed high on the wall at the side of the table and so had a side-on view of the whole scene (mother, infant and the table area in between). This camera was used as a back-up. Infants taking part in this task did so as part of a larger study including other activities not reported here. EEG was also recorded from both mother and infant during the task but is not analyzed here. To control for infants’ language ability, mothers completed the Communicative Development Inventory (Fenson et al., 2007) which provides measures of infants’ early language development.

### Video Coding

Four variables were measured at the trial level, and one at the participant level. Trial level variables were Infant Accuracy (the dependent variable), two measures of infant engagement: Infant Engagement score and Infant Looking During Teaching, and a measure of maternal scaffolding: Maternal Teaching Duration. A further measure of maternal scaffolding, Maternal Sensitivity, was measured at the participant level since mothers tended to maintain a given level of sensitivity across the entire experiment. When coding, the coder noted the start and end times of specific events by video frame (temporal resolution 30 frames per second).

#### Accuracy (Trial Level)

The infant’s accuracy in identifying the bowl where the toy was hidden was both coded live and then checked during video coding (see section “Object Search Task” for details of response categories). Only valid trials where infants clearly searched in the correct or incorrect bowl were included for analysis. This was used as the task outcome measure (dependent variable).

#### Infant Engagement (Trial Level)

Infants’ engagement was scored on a 5-point scale (1 = very low engagement, 5 = very high engagement) based on the infant’s behavior during both the teaching and response phases of the trial. It provided a measure of how engaged the infant was with the goal of the task, i.e., how much he wanted to find the toy. For example, an infant who paid close attention during the teaching phase and was clearly keen to find the toy, straining forward across the table to reach the bowls before the mother had finished pushing them forward, and showing obvious anticipation of finding the toy on lifting one of the cloths (irrespective of whether the toy was successfully found) would score 5, while an infant who paid attention and reached for one of the cloths when offered the bowls but did not show such eagerness in body language might score 4, and an infant who showed no interest in finding the toy and appeared more interested in non-task related activities would score 1. This score was assessed from the video showing only the infant, so although the mother’s voice could be heard, the coder was not aware which bowl contained the toy when the bowls were passed to the infant. Further details of the criteria used to determine this score are given in the Supplementary Material S1.

#### Infant Looking During Teaching (Trial Level)

Infant looking duration was coded to assess the visual attentiveness of the infant during maternal teaching. Looks to mother and/or toy were not differentiated because mothers often held the toy in front of their faces to draw the infant’s attention to it, making it difficult to distinguish which was the focus of attention. Only looks with a duration of more than 0.5 s were included. Raw looking times were affected by the duration of teaching, as trials with longer teaching phases gave the infant more potential looking time. Therefore infants’ looking time was calculated as a percentage of the teaching time.

#### Duration of Teaching (Trial Level)

Mothers’ duration of teaching was measured as starting from the point when she first drew the infant’s attention to the toy and finished when both bowls were covered by cloths. As each mother was at liberty to extend the teaching period until she felt satisfied that her infant understood where the toy was hidden, this was taken as a measure of the mother’s responsiveness to her infant.

#### Maternal Sensitivity Score (Recorded Per-Mother Not Per-Trial)

Each mother was assessed on the extent to which she adjusted her behavior or tone of voice in response to her infant’s signals (Feldman et al., 2009). Attention was particularly paid to how she responded to the infant’s vocalizations, gestures, and periods of fussiness. This was recorded on a 5-point-scale, where a score of 5 indicated that the mother was closely “tuned in” to her infant and always responded, and a score of 1 indicated that the mother paid little attention to her infant’s signals. Judgments were based on how the mother behaved during the task, and also on how she responded between trials (e.g., when retrieving the toys). This measure captured variance in the mother’s *style* of delivery (while Duration of Teaching captured variance in the *pacing* of the mother’s teaching). Further details of the criteria used to determine this score are given in the Supplementary Material S1.

Thus video coding yielded two measures of infant engagement (Infant Engagement Score and Infant Looking During Teaching) and two measures of maternal responsiveness (Mother’s Duration of Teaching and Maternal Sensitivity Score).

As the measures of Infant Engagement and Maternal Sensitivity were more subjective than the timing and accuracy measures, a detailed coding protocol was developed for each to guide the coder in allocating scores in a standardized manner (see Supplementary Material S1). Furthermore, in order to assess the inter-rater reliability of these two measures, seven infants (i.e., 20% of infants in the study) were selected at random and their videos double coded by another coder, blind to the first coder’s decisions, using the same coding protocol. Weighted Cohen’s kappa values for each measure showed good inter-rater agreement (Altman, 1991) for each measure (Infant Engagement: *k* = 0.672; Maternal Sensitivity: *k* = 0.667).

### Data Processing and Analysis

The task comprised 16 trials. Of the 35 infants who took part, 31 completed all 16 trials, and the mean number of trials completed was 15.49 (542 trials in total). When infants failed to complete the full 16 trials, this was because the infant became tired or upset during the task and did not complete the final trial(s). For one infant who completed 16 trials only 8 trials could be video-coded due to a camera error, so this infant only contributed 8 trials to the analysis.

Prior to analysis of the data, 9 trials were excluded where the infant paid no attention to the task at all (i.e., an Infant Engagement score of 1 out of 5) as behavior on these trials would not reflect processes related to object search. Trials were also excluded where the infant did not look at mother or toy during the Teaching Phase at all and so could not possibly know where the toy was hidden (5 trials). Following visual inspection of histograms to identify outlier datapoints, cut-off points were decided for the time the infant took to select a bowl and the duration of teaching such that trials were excluded where the infant took longer than 20 s to select a bowl (eight trials), and where the duration of the Teaching Phase was > 30 s (five trials). Following these exclusions, one infant only contributed 3 trials (which was not sufficiently representative), so this participant was excluded, leaving data from 34 infants in the final analysis, contributing a total of 511 trials. Of the 34 infants in the final analysis, excluded trials were spread across 15 infants, with the maximum excluded trials per infant being 4. The mean number of trials contributed to the analysis was 15.03. In total, exclusions led to the removal of 5.7% of the initial dataset.

Data analysis was carried out by fitting a mixed-effects regression model to the raw data, with random intercepts for participant to account for participant level clustering in the data, using the lme4 package in R (Baayen et al., 2008; Bates et al., 2015). This allowed us to avoid the data loss due to aggregation that comes with calculating participant means, and to investigate how variations in behavior affect performance on *individual trials*, rather than on a per-infant basis. As the dependent variable was binary (*Accuracy*), a generalized mixed-effects model with a logistic link function was used. Since it is not possible to calculate Cohen’s *d* for predictors in a mixed-effects model, marginal *R*^2^, which gives a measure of the variance explained by the fixed effects, was used as a measure of effect size instead (Nakagawa and Schielzeth, 2013). To calculate how much of the variance in the dependent variable was accounted for by each predictor, the marginal *R*^2^ of the full model was compared with the same model that had each predictor removed in turn. Infants’ age and number of words understood (as assessed by the Communicative Development Inventory) were included in every model to control for effects of development. The infant’s age (in days) and the infant looking during teaching (calculated as a proportion of the teaching time) were grand-mean centered to aid interpretation and model estimation.

## Results

Participant means for the variables of interest are shown in Table 2. Infants’ mean accuracy on the object search task (60.82%) was significantly above chance (*t*(33) = 4.241, *p* < 0.001, *d* = 0.73).

**TABLE 2:** Descriptive statistics for the variables of primary interest.

	**Participant mean (SD)**	**Range**
Infant looking during teaching (%)	81.56 (8.89)	54.3–97
Infant engagement (out of 5)	4.36 (0.63)	2.7–5
Mother’s duration of teaching (seconds)	9.33 (3.23)	3.8–15.6
Maternal sensitivity score (out of 5)	4.11 (0.91)	2–5
Infant accuracy (%)	60.82 (14.88)	33–94

### Predictors of Infant Accuracy

Our main aim was to assess whether infants’ accuracy on the task was predicted more strongly by infant engagement or by maternal responsiveness. To assess this, predictors of maternal responsiveness (Mother’s Duration of Teaching and Maternal Sensitivity Score) and infant engagement (Infant Looking During Teaching and Infant Engagement), as well as control variables, were concurrently entered into a model fit to the infant accuracy data as shown in Table 3. The only significant predictor of infant accuracy was the infant’s level of engagement with the task (*p* = 0.010). Infant performance was not related to either measure of maternal responsiveness, or to the infant’s looking during teaching.

**TABLE 3:** Fixed effects from model fit to accuracy data.

	**Estimate**	**SE**	***z***	***R*^2^**	***p***
**Dependent variable: infant accuracy**					
Infant age	< −0.001	0.003	–0.007	0.000	0.994
Infant words understood	<0.001	0.003	0.324	0.000	0.746
Infant looking during teaching	0.004	0.005	0.874	0.002	0.382
Infant engagement	0.300	0.117	2.561	0.019	0.010^∗^
Mother’s duration of teaching	–0.010	0.024	–0.413	0.000	0.680
Maternal sensitivity score	0.166	0.114	1.453	0.006	0.146

*^∗^*p* < 0.05.*

As some of the predictor variables were closely related (particularly Infant Engagement and Infant Looking During Teaching, see Supplementary Material S2), this raised concerns of multicollinearity. Accordingly, we checked for correlations between the variables and conducted further analyses (see Supplementary Materials S2, S3) to confirm that (1) when Infant Engagement was removed from the model, none of the remaining predictors showed a significant relationship with Accuracy; and (2) when any of the other predictors were removed from the model, Infant Engagement remained the only significant predictor of Accuracy.

### Further Analyses

The finding that maternal teaching duration did not relate to infant accuracy was surprising, so we carried out further analyses to investigate whether mothers did in fact adapt their teaching delivery to their children (otherwise the previous null result could simply be attributed to a lack of variance in maternal behavior). The finding that Maternal Sensitivity Score did not affect accuracy was less surprising as this was not measured for each individual trial and therefore was expected to have less effect on individual trial performance. First, we asked whether the variance in teaching time was due to mothers adapting their teaching on individual trials, or simply due to differing teaching styles where some mothers would routinely teach for longer times, and some for shorter times, with little inter-trial variation. The mean teaching time (by participants) was 9.3 s, but the mean difference between each mother’s longest and shortest teaching time was 11.8 s, showing that mothers were adjusting their teaching times on individual trials.

To examine whether maternal teaching time varied significantly in accordance with infant behavior, we fit a regression model with Mother’s Duration of Teaching as the dependent variable. This showed that mothers significantly extended the teaching time when the infant was younger, and when the infant looked less during the teaching period (Infant Age: β = −0.032, St Error = 0.015, *R*^2^ = 0.053, *t* = −2.122, *p* = 0.042; Infant Looking During Teaching: β = −0.066, St Error = 0.008, *R*^2^ = 0.072, *t* = −8.155, *p* < 0.001). This result confirmed that mothers did indeed adapt their delivery of the task in accordance with their infants’ age and visual attention to the task. The infant’s receptive vocabulary, Infant Engagement and Maternal Sensitivity Score were also included in the model but were not significant predictors. A similar regression examining predictors of Maternal Sensitivity Score showed no significant relationships with predictor variables.

Having confirmed that mothers significantly varied their teaching duration in response to their infants, we carried out two further analyses to assess the effect of such maternal modulation on infant performance. First, since teaching times were significantly longer on trials where the infant paid less visual attention to the task, we examined performance on shorter and longer looking trials separately to see whether longer teaching times conveyed any advantage (section “Effects of Maternal Teaching Duration on Shorter/Longer Looking Trials”). If infants paid little visual attention to the game because they were distracted, they would be predicted to benefit from their mother’s extended teaching and attentional direction toward task-relevant information. However, if infants were inattentive because they already grasped early on where the toy was hidden, they would have no further need for maternal elaboration, and so might perform worse when their mother lengthened the teaching time, which could increase infants’ boredom. Secondly, we examined perseverative errors, as these are considered the most common type of error in these kinds of task (Diamond, 1988), asking whether the mother’s duration of teaching or sensitivity score had a stronger predictive value for trials where infants successfully overcame a perseverative response (section “Factors Affecting Perseverative Search Patterns”).

#### Effects of Maternal Teaching Duration on Shorter/Longer Looking Trials

**FIGURE 2:**
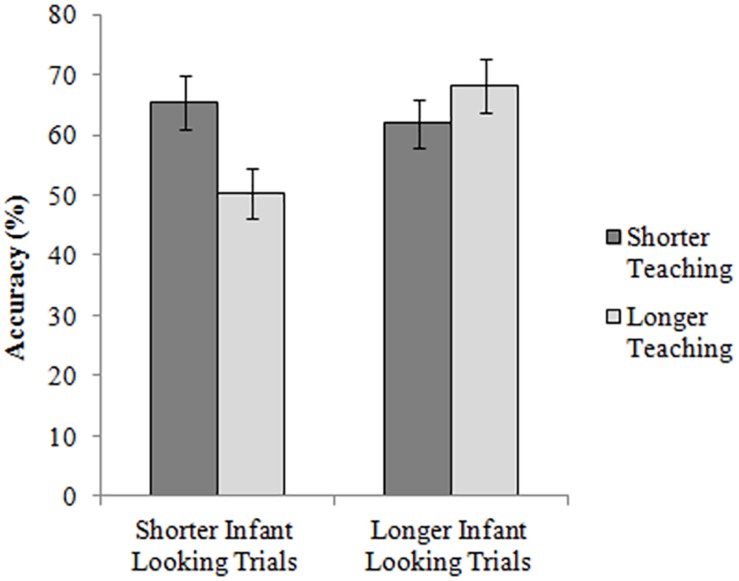
Accuracy levels for trials divided by duration of infant’s looking during teaching, and duration of teaching time. Error bars show one standard error.

Trials were divided into two groups (median split) depending on the infant’s looking time. Within each of these two groups, trials were divided into those with longer and those with shorter teaching times (median split). As Figure 2 shows, for trials with shorter looking times, accuracy was significantly *lower* when the teaching time was extended (65.5 vs. 50.4%, *t*(245.367) = 2.463, *p* = 0.014, *d* = 0.318), whereas for trials with longer looking times the duration of teaching had no significant effect on accuracy (62.0 vs. 68.1%, *t*(244.202) = −0.1.027, *p* = 0.305, *d* = 0.129). This suggests that, for trials with shorter infant looking times, mothers’ elaboration of teaching was associated with *worse* performance.

#### Factors Affecting Perseverative Search Patterns

To investigate factors affecting the classic perseverative search pattern, performance on trials where the infant looked for the toy on the same/different side to where he had looked in the previous trial was compared, as shown in Table 4. The first trial for each infant, and any trial following an invalid response, were excluded. In line with previous literature regarding infant’s tendency to show patterns of perseveration, infants showed a preference for searching for the toy on the same side as they had searched in the previous trial (298 out of 511 trials = 64% of trials). However, as shown in Table 4, when infants were able to overcome a perseverative pattern of searching, their accuracy was significantly higher for these different-side-search trials than for same-side-search trials (*t*(389.678) = −5.655, *p* < 0.001, *d* = 0.535).

**TABLE 4:** Performance on trials where infant searched on same/different side as on the previous trial.

	**Infant searches on same**	**Infant searches on different**
	**side as previous trial**	**side from previous trial**
Number of trials	298	163
Accuracy (%)	54.03 (49.92)	78.53 (41.19)

*Standard deviations are shown in brackets.*

As infants showed the highest accuracy on different-side-search trials, we asked whether the superior performance on these non-perseverative trials was explained by maternal scaffolding or infant internal factors. A mixed-effects model fit to the subset of different-side-search trials in the same way as for the main analysis showed that the only significant predictor of Accuracy was (again) Infant Engagement (β = 0.607, St. Error = 0.306, *z* = 1.981, *p* = 0.048). The same model fit to the subset of same-side-search (i.e., perseverative) trials showed no significant predictors. Thus, we found no evidence that the infant’s ability to break the perseverative pattern was associated with the mother’s performance.

## Discussion

In order to assess whether infant performance on a naturalistic object search task was more strongly affected by maternal scaffolding (modifications in mothers’ behavior in response to infants) or infant internal factors (such as the infant’s engagement in the task), infants and their mothers took part in a hiding and finding game where the mother hid a toy in one of two covered bowls for the infant to find. The task was delivered in a naturalistic game-playing manner.

The main analysis showed that the infant’s success on the task (i.e., looking for the toy in the correct bowl) was predicted only by the infant’s task engagement, and not by the infant’s looking patterns, the duration of the mother’s teaching, or the mother’s sensitivity during their social interaction.

To investigate the negative finding that infant’s performance was not related to the mother’s behavior, further analyses were carried out. We showed that although mothers varied their teaching time from trial to trial in response to their infants’ age and perceived attentional status, such maternal modulation had the opposite effect to what was intended: on trials where the infant paid little visual attention to the task, extended maternal teaching resulted in *lower* accuracy compared to trials where maternal teaching was kept brief. For trials where infants successfully overcame a perseverative bias, performance was again predicted only by the infant’s engagement with the task, and not by either the mother’s teaching time or her sensitivity score.

Our findings support predictions made by the infant internal hypothesis and suggest that, in this particular task, the infant does not derive positive benefit from maternal scaffolding (in terms of lengthening the teaching time), indeed, he may even be hindered by maternal elaboration. Rather, infant performance on this task was primarily driven by internal factors relating to engagement. In the following section we consider possible reasons for this result.

As mothers spent a longer time teaching on trials where the child paid less visual attention to the task, it might be suggested that it is this lack of attention that predicts poor performance, rather than maternal teaching *per se*. However, in our main model, *Infant Looking during Teaching* was *not* a significant predictor of infants’ Accuracy, nor did it emerge as a significant predictor even after Infant Engagement was removed from the model (Supplementary Material S3).

Although infants’ performance on this task was not affected by the amount of time the mother spent teaching the location of the hidden object, this is not to say that infants do not benefit in general from such maternal adaptations. Research on maternal sensitivity and responsiveness has shown that responsive parenting confers many advantages for a child (e.g., Landry et al., 1997; Tamis-LeMonda et al., 2001; Paavola et al., 2005; Bornstein et al., 2008; Wass et al., 2018b), so although we do not see a direct effect here it may be that the cumulative effects of responsive parenting are only apparent in the longer term. Similarly, during interactive play, parents use social cues to scaffold their infant’s attention patterns, leading to the infant showing more adult-like attention patterns over time (Bibok et al., 2009; Dilworth-Bart et al., 2010; Wass et al., 2018a, b). However, at ten months of age, infants may not yet have had time to benefit from such scaffolding or they may be unable to make use of social information in the immediate context.

A further possible explanation for the lack of effect of maternal teaching duration on infant performance may be a misreading by the mother of the child’s behavioral cues. It could be that when looking away, the infant is signaling that he already has the information needed. However, mothers may misinterpret this behavior as distraction and continue trying to engage the infant in the task. Since infants were shown four demonstration trials (of the toy being hidden and found by the mother) prior to taking part, more advanced infants may well have realized that only a quick glance at the right moment was required to see where the object was hidden. In cases where the infant quickly assimilated the location of the toy and then looked away, prolonged teaching on the part of the mother could have led to an inaccurate response (see Figure 2) either through boredom induced by the mother’s attempts to re-engage him/her, or due to the increased memory load induced by the delay between first seeing the toy being hidden and being asked to find it.

Alternatively, it may be that, as Topál et al. (2008) propose, it is the infants who misinterpret their mother’s communicative efforts, assigning the taught location as a property of the toy, rather than as episodic information about a temporary hiding place. In Topal’s paradigm infants were trained to find the toy at location A (four A trials) before the toy was then hidden at location B for three B trials. In contrast, in our study the hiding location mostly alternated from side to side, effectively making most trials “B trials” that followed a single “A trial.” Because of the reduced training on A trials in our task, the “location-as-a-property-of-the-toy” interpretation seems less likely for our data. However, if it is the case that infants are interpreting the hiding location as a property of the toy, it would follow that the more effectively the mother demonstrates the (constantly changing) hiding location of the toy, the more confused the infant might become about where to look for it. Recent work supports the suggestion that an infant’s attention is less affected by social cues from an adult play partner than previously thought. Infant’s longer looking times to toys during joint attention periods had been interpreted as showing that infants showed better endogenous attention control in social contexts (Yu and Smith, 2016). However, Wass et al. (2018a) suggest that these longer looking times may be explained by bottom-up factors such as the increased saliency of a toy when it is being manipulated by an adult.

As mothers varied their teaching times considerably from trial to trial, and their teaching time for each trial was predicted by the infant’s looking time, an interesting question to consider is *why* mothers varied their performance in this way. The relationships observed in the data do not allow us to distinguish between a scenario in which the mother increases her teaching time because her infant seems inattentive, and one in which the infant pays less attention as a result of prolonged teaching. However, it seems reasonable to speculate that at least part of the effect is due to the mother’s assumption that further teaching will assist her inattentive infant in successfully finding the toy, an assumption which our results suggest is misguided.

There were several limitations to the current study. The sample size of 35 infants was relatively small, and the measures of maternal scaffolding were limited to the mother’s duration and style of teaching. In further work it would be interesting to investigate the role of maternal presentation style more comprehensively by assessing other measures such as mutual gaze, use of the infant’s name, parental playfulness or synchronicity. Such future studies should also use a more quantitative measure of maternal sensitivity. It would be interesting to manipulate parametrically the length of the teaching time to see whether accuracy is improved when the teaching time is kept short, compared to longer, or infant adaptive teaching times. Further work should also look more comprehensively at infant behaviors, particularly the development of a more quantitative measure of Infant Engagement, with the aim of understanding how different aspects of engagement drive performance. An additional question for future exploration would be whether similar results are found when the infant is interacting with a stranger rather than his/her mother. Previous research has shown that eight-month-old infants show stronger gaze-following with strangers than their mothers (Gredebäck et al., 2010), and it may be that this unfamiliarity effect would lead to differing behavior patterns.

In sum, while parental influences are no doubt crucial to an infant’s development over longer time-scales, it seems that in certain tasks, at a trial-to-trial timescale, it is the child’s endogenous engagement that determines success, despite adaptations made by mothers on behalf of their children. This perspective may be useful for parents to bear in mind, i.e., that in certain contexts and over short time-scales, their infant’s performance may depend in larger part on internal factors rather than parental influences.

## Data Availability Statement

The datasets generated for this study are available on request to the corresponding author.

## Ethics Statement

This study was carried out in accordance with the recommendations of Cambridge Psychology Research Ethics Committee with written informed consent from all subjects. Parents gave written informed consent on behalf of their children in accordance with the Declaration of Helsinki. The protocol was approved by the Cambridge Psychology Research Ethics Committee (PRE.2016.029).

## Author Contributions

VL, SW, KC, and SG designed the study. VL, KC, SG, LB, and RN collected the data. KC, HA, JB, GC, and BR carried out video coding and KC analyzed the data.

## Conflict of Interest

The authors declare that the research was conducted in the absence of any commercial or financial relationships that could be construed as a potential conflict of interest.

## Footnotes

1 For a linear multiple regression random effects model with up to seven predictors (ρ^2^ = 0.35), a sample size of *N* = 32 provides power of 0.72 at α = 0.05.
