# Supplementary material for: Do Helpful Mothers Help? Effects of Maternal Scaffolding and Infant Engagement on Cognitive Performance
Source: Front Psychol. 2019 Nov 28;10:2661. doi: 10.3389/fpsyg.2019.02661 (PMC6896844; doi:10.3389/fpsyg.2019.02661)
Supplement: Supplementary file 1 [file Data_Sheet_1.pdf]

Supplementary Materials

S1 Criteria used to determine levels of subjective variables

Infant Engagement Score

The Infant Engagement score sought to reflect the degree to which the infant engaged with the goal of the task, i.e. how much he wanted to find the hidden toy. For each trial, the coder watched the video of the infant during the teaching and response phases. Although the coder could hear the mother's teaching, the video showed only the infant, so the coder was not aware which bowl contained the toy when the bowls were passed to the infant.

The coder then allocated the trial an Infant Engagement score between 1 and 5. A score of 1 reflected a trial in which the infant had no interest in the task at all, while a score of 5 reflected a trial where the infant showed a strong desire to find the toy (irrespective of whether the infant was successful in finding the toy).

In allocating a score, the coder considered:

- *Affect.* The infant's general mood/level of fussiness before the trial and how the infant responded to the initiation of a new trial. An infant who showed negative affect or no interest in or reaction to the start of the trial would get a low score, whereas an infant who showed positive affect and clearly responded positively when the mother held up the toy to be hidden (even if previously the infant had been fussy and upset) would get a high score.
- *Interest.* The degree to which the infant appeared focussed on where the mother was placing the toy. The timing of the hiding of the toy was clear from the audio of the

mum saying "look, I'm putting this in here" or similar. An infant who showed no particular interest in the hiding of the toy would get a low score, while an infant who focused intently on the task at this crucial point would get a high score. (Mothers were instructed not to continue with the task unless the infant had seen where the toy was hidden, but this varied from infants tracking maternal actions closely to infants glancing at the object in passing.)

- *Body language*. An infant who sat back in his chair, looking around him or playing with non-task objects (e.g. high chair straps, or his socks) would get a low score, whereas an infant whose body was oriented towards the task and who reached forward across the table for one of the cloths even before the bowls were in reach would get a high score.
- *Goal-directedness.* This was exemplified by the manner in which the infant interacted with the experimental apparatus. An infant who pulled the cloth off the bowl but then waved it around, seemingly uninterested in whether there was anything in the bowl would get a lower score than an infant who very deliberately grabbed at a cloth and looked in the bowl for the toy.

To validate the Engagement Score, we tested whether Infant Engagement scores predicted the infant's response time (i.e. the time between the infant being presented with the two bowls and the time at which he touches one of the cloths), which was indeed the case (β=-0.263, Std Error=0.112, t = -2.355, p = 0.019). Reaction time is a recognised measure of cognitive engagement (i.e. monitoring or vigilance; Buck, 1966), and it is an objective index that does not rely on subjective coding. Since reaction time is correlated to infant engagement, this supports the view that both indices tap into similar mental processes. It

is also possible that reaction time and engagement scores co-vary because of a third factor, but in our view this is less likely.

Maternal Sensitivity Score

The Maternal Sensitivity score provided a measure of how often the mother responded to her infant's signals or adjusted her behaviour or tone of voice in response to her infant. For each mother/infant pair, the coder watched the video of the whole session (taken from the side, so both mother and infant were visible). The task instructions made it clear to the mother that the task should be delivered in a naturalistic, engaging, game-playing manner, and that the object was to make it fun for the baby, so despite the clearly outlined steps of the task, mothers had considerable flexibility in their manner of delivery.

The coder allocated the mother a Maternal Sensitivity score between 1 and 5. A score of 1 reflected a mother who ploughed on with the task paying no heed to her infant's mood or bids for attention, while a score of 5 reflected a mother who was 'tuned in' to her infants needs, continually watching for the infant's signals and adjusting her behaviour in response.

In allocating a score, the coder considered:

- Examples of imitation such as the infant smiling and the mother smiling back, or the mother responding to a noise from the infant by copying the infant's noise, or making a noise in response. A mother who consistently responded to her child's bids for attention with imitation or other responses would get a high score, whereas a mother who routinely ignored her infants vocalisations or gestures would get a low score.

- The extent to which (within the confines of the task instructions she had been given) the mother adjusted her behaviour in response to the infant's mood, such as responding to gestures, crying or infant fussing. A mother who ignored her infant's changes in mood and simply delivered the task would get a low score. A mother who responded sympathetically when the infant was upset, e.g. pausing in her delivery of the task and saying "You poor little thing, you're getting tired, aren't you?" in acknowledgement of the infant's cries would get a high score. Similarly, a mother who responded to her infant pointing at the toy by saying "Yes, you like this train, don't you? Look where I'm going to hid it." would get a higher score than a mother who ignored her infant's gesture.
- Instances where the mother praised or commented on the infant's performance. A mother who made no comment on the infant's performance on the task would get a low score, while a mother who commented on how her infant responded to being presented with the bowls (irrespective of whether the successfully found the toy) would get a higher score. For example, a mum might say "You think it's in that one do you?" as her infant reached for one of the cloths, or "Good try, where do you think it is?" when her infant looked in the empty bowl.
- How the mother interacted with the infant between trials (when she was freed from the specific task instructions she had been given). If the baby was becoming fussy and the mother simply carried on with the next trial she would get a low score, whereas a mother who took a bit of time to cheer up her fussy infant between trials, such as by reaching across the table to hold the infant's hands and establish eye contact would get a higher score.

- How the mother took the toy back from her infant ready for the next trial. Infants were often reluctant to give up the toy once found, and mothers varied widely in the strategies they used to encourage the infant to return the toy, and to deal with their child's reluctance to hand over the toy. Some would be impatient to get the toy back and start the next trial, even if this meant upsetting the infant who wanted to continue playing with the toy (low score), whereas others would wait until the infant was ready to hand over the toy, or use distraction techniques to remove the toy without upsetting the infant (high score).

S2 Variable correlation matrix

The correlation matrix showed a number of correlations between predictor variables and infant Accuracy.

	Infant Looking During Teaching	Infant Engagement	Mother's Duration of Teaching	Maternal Sensitivity Score
Infant Engagement	.331**			
Mother's Duration of Teaching	-.283**	-.117**		
Maternal Sensitivity Score	0.023	.106*	-.111*	
Accuracy	.092*	.147**	-0.068	.091*

Supplementary Materials Table 1: Correlation matrix of predictors and outcome measure from main analysis. Analyses carried out on data at trial level: degrees of freedom = 509. (* indicates p<.05; ** indicates p<.001)

S3 Confirmatory regression analyses

To explore the possibility that the effect of Infant Engagement might be masking the effect of another predictor variable, each predictor was removed from the regression model (shown in Table 3 of the main manuscript) in turn in order that any effects of the remaining variables might be seen:

When Infant Engagement removed from Model:

	Estimate	Std. Error	*z*	*p*
(Intercept)	-0.240	0.519	-0.462	0.644
Infant Age	0.000	0.003	0.151	0.880
Infant Words Understood	0.000	0.003	0.096	0.923
Infant Looking During Teaching	0.008	0.005	1.740	0.082
Mother’s Duration of Teaching	-0.012	0.024	-0.496	0.620
Maternal Sensitivity Score	0.194	0.109	1.771	0.077

When Infant Looking During Teaching removed from model:

	Estimate	Std. Error	*z*	*p*
(Intercept)	-1.512	0.721	-2.096	0.036
Infant Age	0.000	0.003	-0.112	0.911
Infant Words Understood	0.001	0.003	0.298	0.766
Infant Engagement	0.327	0.112	2.915	0.004*
Mother’s Duration of Teaching	-0.016	0.023	-0.720	0.471
Maternal Sensitivity Score	0.163	0.113	1.443	0.149

When Mother’s Duration of Teaching removed from model:

	Estimate	Std. Error	*z*	*p*
(Intercept)	-1.587	0.676	-2.348	0.019
Infant Age	0.000	0.003	0.100	0.920
Infant Words Understood	0.001	0.003	0.383	0.701
Infant Looking During Teaching	0.005	0.005	1.052	0.293
Infant Engagement	0.304	0.118	2.583	0.010*
Maternal Sensitivity Score	0.169	0.116	1.450	0.147

And with Maternal Sensitivity Score removed:

	Estimate	Std. Error	*z*	*p*
(Intercept)	-0.901	0.598	-1.508	0.131
Infant Age	0.000	0.003	0.112	0.911
Infant Words Understood	0.002	0.003	0.538	0.591
Infant Looking During Teaching	0.004	0.005	0.856	0.392
Infant Engagement	0.323	0.118	2.743	0.006*
Mother’s Duration of Teaching	-0.010	0.025	-0.395	0.693

* = $p<.005$

References

Buck, L. (1966). Reaction time as a measure of perceptual vigilance. Psychological Bulletin, 65(5), 291-304.
